# Updated approach for the management of osteoporosis in Turkey: a consensus report

**DOI:** 10.1007/s11657-020-00799-0

**Published:** 2020-08-29

**Authors:** Yeşim Kirazlı, Funda Atamaz Çalış, Özlem El, Yeşim Gökçe Kutsal, Özlen Peker, Dilsad Sindel, Şansın Tuzun, Dilek Gogas Yavuz, Berrin Durmaz, Ülkü Akarirmak, Hatice Bodur, Vedat Hamuryudan, Umit Inceboz, Sema Öncel

**Affiliations:** 1 Department of Physical Medicine and Rehabilitation, Medical Faculty of Ege University, Izmir, Turkey; 2 Department of Physical Medicine and Rehabilitation, Faculty of Medicine, Dokuz Eylül University, İzmir, Turkey; 3 Department of Physical and Rehabilitation Medicine, Hacettepe University Medical School, Ankara, Turkey; 4 Department of Physical Medicine and Rehabilitation, Istanbul Faculty of Medicine, Istanbul University, Istanbul, Turkey; 5 Department of Physical Medicine and Rehabilitation, Cerrahpaşa Medical Faculty, Istanbul University Cerrahpaşa, Istanbul, Turkey; 6 Department of Internal Medicine, Section of Endocrinology and Metabolism, Marmara University School of Medicine, Istanbul, Turkey; 7 Department of Physical Medicine and Rehabilitation, Medical Faculty of Yıldırım Beyazıt University, Ankara, Turkey; 8 Department of Internal Medicine, Section of Rheumatology, Cerrahpaşa Medical Faculty, İstanbul University Cerrahpaşa, İstanbul, Turkey; 9 İrenbe Obstetrics and Gynecology IVF Center, İzmir, Turkey

**Keywords:** Diagnosis of osteoporosis, Treatment of osteoporosis, Fracture risk assessment, FRAX

## Abstract

***Summary*:**

As a result of the current demographics, increased projections of osteoporosis (OP) and prevalence of the disease in Turkey, a panel of multidisciplinary experts developed a thorough review to assist clinicians in identifying OP and associated fracture risk patients, diagnosing the disease with the appropriate available diagnostic methods, classifying the disease, and initiating appropriate treatment. The panel expects to increase the awareness of this prevalent disease, decrease consequences of OP with corresponding cost savings and, ultimately, decrease the overall burden of OP and related fractures in Turkey.

**Background:**

OP is not officially accepted as a chronic disease in Turkey despite the high prevalence and predicted increase in the following years. However, there are areas where the country is performing well, such as having a country-specific fracture risk assessment model, DXA access, and the uptake of FRAX. Additional efforts are required to decrease the existing treatment gap estimating 75–90% of patients do not receive pharmacological intervention for secondary prevention, and the diagnosis rate is around 25%.

**Methods:**

A selected panel of Turkish experts in fields related to osteoporosis was provided with a series of relevant questions to address prior to the multi-day conference. Within this conference, each narrative was discussed and edited by the entire group, through numerous drafts and rounds of discussion until a consensus was achieved. Represented in the panel were a number of societies including The Turkish Osteoporosis Society, The Society of Endocrinology and Metabolism of Turkey (SEMT), and The Turkish Society of Physical Medicine and Rehabilitation.

**Results:**

Standardized general guidelines to identify OP and related fractures and at-risk population in Turkey, which will enable clinicians to accurately and effectively diagnose the disease, treat the appropriate patients with available pharmacological and non-pharmacological treatments and decrease the burden of the disease.

**Conclusions:**

This manuscript provides a review of the current state of OP and related fractures in Turkey. Moreover, this manuscript reviews current international guidelines and national studies and proposes a number of helpful country-specific classifications that can be used by healthcare providers caring for the at-risk population. Additionally, the panel proposes practical recommendations that should be implemented nationally in order to decrease the burden of OP and related fractures and effectively preventing the burden in future generations.

Osteoporosis (OP) is defined as a systemic disorder characterized by low bone mass and microarchitectural deterioration of bone tissue with a consequent increase in bone fragility and susceptibility to fracture [1]. OP and related fractures are becoming a global epidemic as a result of an aging population with a longer life span. Therefore, OP has been identified as a “global health problem” by the World Health Organization (WHO) [2, 3]. Increased global socioeconomic burdens of OP-related fractures make the prevention of such injuries a major public health goal with an estimated savings of up to 50% of all hip fracture expenditures [4, 5].

## Materials and methods

To address the above issues, the Americas Health Foundation (AHF) identified relevant OP societies in Turkey and determined the associated clinicians and scientists with an academic or hospital affiliation who are experts in the field and who have published in the OP arena since 2012. As a result of this effort, AHF convened an eight-member panel of clinical and scientific experts from Turkey. Great attention was paid to ensure a diverse group representing various disciplines related to OP.

To better focus on the discussion, AHF staff independently developed specific questions, addressing the salient issues on the subject, for the Panel to address. A written response to each question was initially drafted by a different member of the Panel. During the multi-day meeting of the Panel, each narrative was discussed and edited by the entire group, through numerous drafts and rounds of discussion until complete consensus was obtained. The objective of this article is to create a practical document with standardized guidelines for counseling, screening and diagnosing OP in Turkey.

### Search strategy and selection criteria

Specific questions, addressing the salient issues on the subject, were sent to the panel members to address. A written response to each question was initially drafted by individual members of the Panel. Manuscripts referenced in this consensus paper were identified through searches of Pub Med and Embase with the search terms “osteoporosis”, “osteoporosis in Turkey”, “hip fractures”, “diagnosis of osteoporosis,” and “treatment of osteoporosis” from July 2014 to July 2019, and the list of the references were sent to the panel members before the multi-day meeting of the panel. Additionally, throughout the meeting, the panelists had the opportunity to add literature as well as sources of their own files. Particular attention was paid to papers that reviewed or summarized the topic in question or that were related to activities in Turkey. The final reference list was generated on the basis of the relevance to the broad scope of this consensus document during the multi-day meeting of the Panel.

Currently, more than 200 million people worldwide are estimated to be osteoporotic. Fragility fractures caused by minimal trauma are the most important clinical outcome of OP [6]. More than one in three postmenopausal women and approximately one in five men over the age of 50 will eventually experience osteoporotic fractures [7]. The most common fracture sites include the vertebral bodies, proximal femur, distal forearm, and proximal humerus, in order of frequency. Worldwide, an osteoporotic fracture is estimated to occur every 3 s, a vertebral fracture every 22 s. Vertebral and hip fractures are highly associated with morbidity and mortality. In the case of hip fracture, 20–30% of the patients die in the first 3–6 months [8]. Vertebral osteoporotic fractures, although mostly asymptomatic, are three times more frequent than hip fractures. The age-adjusted relative risk of dying following a hip fracture is 6.68 and following a vertebral fracture is even higher at 8.64 [9].

Compared with other osteoporotic fractures, hip fractures require special attention given the high mortality risk associated. The expected risk of death for a woman with a hip fracture is 10–20% higher than that of her female peers. A large increase in the number of hip fractures is expected in Turkey within the next 20 years as a result of an aging population. Currently, 8.7% of the Turkish population is over 65 years of age, and this percentage is projected to increase to 16.5% by 2040 [10]. Since hip fracture risk increases exponentially with age, there will be a large significant increase in the number of hip fractures in the country.

The FRACTURK study demonstrated that although 73% of hip fractures occurred in women over 75 years of age, hip fracture rates were similar in men and women between the ages of 50 and 64 years, as the lifetime probability of sustaining a hip fracture at 50 years of age was 15% in women and 3.5% in men. Additionally, this study showed that the average 10-year probability of sustaining a hip fracture increased with age. A 30-year age increase can raise the risk of hip fracture by 3.4% in men and 7.0% in women [11]. According to the International Osteoporosis Foundation (IOF) hip fracture map, Turkey is considered a high-risk country for hip fractures in women [12].

## Risk factors for OP and related fractures

The prevalence of OP increases progressively with age, and the overall prevalence in men and women aged 50 years or more is calculated for Turkey as 22.2 and 27.2%, respectively [13], which makes OP a highly probable disease to be found when actively questioning patients within the age frame about signs or symptoms. However, when assessing OP and fracture risk, measurement of bone mineral density (BMD) will be required but has specific indications (Table 2) that will complete the diagnosis and guide the management of the patient [14]. Risk factors for OP fractures include the following: age, gender, race, geographical region, genetics, diet, lifestyle, hormonal status, body mass index, and other medical comorbidities. Additionally, falls are important and preventable complications that are due to a number of risk factors such as: lack of physical activity, muscle weakness, gait and balance problems, neuromuscular diseases, disability of the lower extremities, or impaired proprioception [15, 16]. All these risk factors for OP and fractures, should be considered and addressed when managing a patient with suspected or diagnosed bone loss [17].

In daily clinical practice, doctors also assess a patient’s risk factors for fracture rather than relying on BMD values alone to decide whether a person should be referred for treatment. Risk of fracture can be assessed by the use of fracture risk algorithms (FRAX). FRAX is a computer-based algorithm that estimates an individual’s 10-year probability of a major fracture and should be used routinely when identifying one of the aforementioned risk factors in patients [18].

**Fig. 1:**
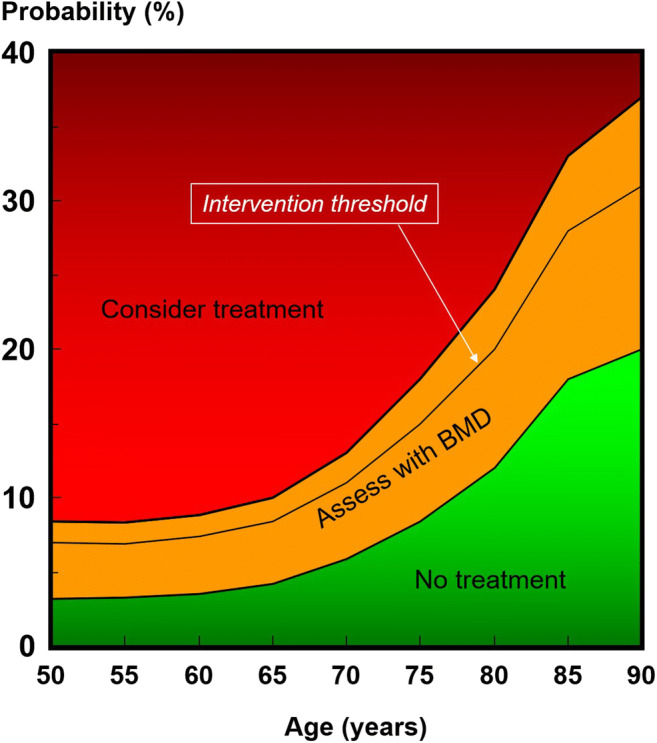
Ten-year probability of a major fracture (in percent) at an intervention threshold and the upper and lower BMD assessment thresholds in women. Body mass index was set to 30.9 kg m^−1^

The Turkey-specific FRAX model was developed to assess fracture risk in patients in the country [13]. The intervention threshold is set at the age-specific fracture probability equivalent for women with a prior fragility fracture. Figure 1 gives the age-specific upper and lower assessment thresholds for risk evaluation and intervention. Treatment can be recommended without the requirement of BMD tests in individuals with a major fracture probability that exceeds 10%. Turkish women eligible for intervention also included those with a prior fragility fracture, comprising 8.6% of women aged 50 years or more. In total, 23.3% of women would be eligible for treatment.

## Diagnosis of OP

There are a number of guidelines used to assess patients with OP [1, 19–23]. To diagnose OP, a detailed medical history, complete clinical examination, and the assessment of fracture risk using a FRAX are recommended. Definite diagnosis of OP is based on the presence of a low-energy fracture and/or the measurement of BMD [19, 21]. The WHO created a scale to determine the level of bone density shown by the T score based on BMD [24]. (Table 1).

**Table 1:** Diagnostic criteria of osteopenia/osteoporosis based on dual-energy X-ray absorptiometry (DXA)

T score	Definition
≥ − 1.0	Normal
− 1.0 to − 2.5	Low bone mass (osteopenia)
≤ − 2.5	Osteoporosis

Source: WHO Study Group [24]

OP cannot be diagnosed on the basis of BMD solely in premenopausal women, men under 50 years of age, and children [22]. T score and the WHO classification of BMD are used to diagnose osteoporosis in postmenopausal women and men over 50 years of age. Based on the most important published guidelines, this panel recommends the following indications for BMD measurement (Table 2).

**Table 2:** Bone mineral density measurement indications

• All women aged 65 and above and all men aged 70 and above • Perimenopausal and postmenopausal women aged below 65 and men aged 50–69 having risk for fracture ○ Low body weight (body mass index < 20 kg m^−2^) ○ Long-term systemic glucocorticoid treatment (≥ 3 months) ongoing or started recently ○ Family history of osteoporotic fracture ○ Early menopause (< 45 years of age) ○ Smoking ○ Excessive alcohol consumption • Adults having fragility fracture after 50 years of age • Secondary osteoporosis^a^	

^a^Table 3 describes the causes of secondary OP

Z score testing is used in premenopausal women, men under 50 years of age and children and it compares bone density with the average bone density of a similar age and gender population. Although the International Society for Clinical Densitometry (ISCD) suggests using Z score in premenopausal women, IOF recommend Z scores only in children and adolescents. A Z score < − 2.0 is interpreted as “lower than the expected interval” with regard to age and indicates causes of secondary OP that should be studied [21].

### BMD measurement

BMD measurement is important in OP screening as it will help determine fracture risk in individual patients and identify appropriate candidates for pharmacological treatment. BMD is also useful in the follow-up of treated and untreated patients [22, 25]. Indications for BMD measurement have been listed in Table 2. DXA is the standard method to measure BMD.

The skeletal sites of lumbar spine, proximal femur, and when needed, 1/3 distal radius measurements should be considered when analyzing BMD by DXA. This panel supports central DXA (lumbar spine and proximal femur) measurement recommendations in the diagnosis of OP defined by ISCD [22]:

- Both posteroanterior spine and hip measurements should be performed
- Forearm BMD measurement can be used when hip and/or spine measurements cannot be done, the measurement cannot be interpreted due to severe degenerative disease and widely used surgical instrumentation, presence of hyperparathyroidism, and, in extremely obese patients with weight and size exceeding the limits of DXA table
- Posteroanterior L1–L4 should be used for BMD measurement of the spine
- All measurable vertebrae should be used excluding those with local structural changes
- Diagnostic classification based on BMD should not be made using only one vertebra
- Anatomically abnormal vertebrae, vertebrae which cannot be assessed precisely or those having a T score difference over 1.0 with the neighboring vertebrae may be excluded
- Lateral spine should not be used for diagnosis
- The lower of the two T scores, femur neck or total proximal femur, is used in diagnosis BMD measurement of any one of the two hips may be performed

Repeat BMD measurements can be performed in specific cases: to support the decision to initiate treatment for untreated patients in the presence of significant bone loss, to evaluate response to treatment in treated patients, to re-evaluate therapy or to identify causes of secondary OP in patients not responding to treatment. Frequency of BMD measurements is determined according to the clinical condition of each patient. However, the recommendations of this panel to complete repeat BMD measurements are (1) in untreated or low-risk patients, BMD should be performed at least every 2 years; (2) for high-risk OP patients, BMD should be performed at least every year as long as the risk persists; and (3) for patients in conditions associated with rapid bone loss, such as glucocorticoid treatment, measurements may be performed in more frequent intervals.

BMD measurement by DXA that is performed with different devices are not comparable, therefore, follow-up should always be performed with the same DXA device. Precision assessment according to standard methods should be made and the least significant change (LSC) should be calculated in order to verify that the change in BMD is true and reliable [22].

### Trabecular bone score

Trabecular bone score (TBS) has become a useful complementary tool for osteoporosis risk classification [26, 27]. A low TBS is independent of BMD, is associated with fracture history and can help assess the risk of a new fracture. TBS should not be used alone to determine treatment recommendations; however, it can be used in postmenopausal women and elderly men for identifying fracture risk in relation to FRAX and BMD [27]. At the same time, TBS is important in the evaluation of fracture risk in patients with secondary osteoporosis such as patients with type 2 diabetes, primary hyperparathyroidism or subclinical hypercortisolism. But, TBS is influenced by adiposity, and this is accounted for in the current software. Only the latest version of TBS adjusts for it. If there is an inconsistency between BMD values of the lumbar vertebrae and hip, the fracture risk can be evaluated using TBS [27].

### Other methods used for BMD

In case DXA is not appropriate for diagnosis, alternative measures include quantitative computed tomography (QCT), peripheral quantitative computed tomography (pQCT), quantitative ultrasound (QUS) and peripheral dual-energy X-ray absorptiometry (pDXA). Peripheral bone density measurements are important in detecting increased fracture risk. Nevertheless, only axial and distal $$ \raisebox{1ex}{$1$}\!\left/ \!\raisebox{-1ex}{$3$}\right. $$ radius measurements are used as diagnostic DXA criteria [21, 24]. It is stated that other technologies should not be used in the diagnosis of OP but they can be used in the assessment of risk fractures [21].

QCT is sensitive in determining vertebral bone loss, in monitoring of the effects of therapy, and imaging in the presence of spinal disease or artifact. pDXA and QUS are frequently used in public-based screening programs since the equipment is portable and accessible, even though these are not indicated as diagnostic techniques for OP given the fact that if abnormal results are detected with these techniques, they should be verified by physical examination, risk assessment and central DXA [1].

### Vertebral imaging

In clinical practice, lateral thoracic and lumbar spine x-ray or densitometric vertebral fracture assessment (VFA) should be performed in patients with unexplained back pain or height loss. X-ray images should not be used for the diagnosis of OP in patients other than those with suspected vertebral fractures [21]. This panel recommends the following indications for vertebral imaging:

- All women 70 years of age or over and all men 80 years of age or over with T score values ≤ − 1.0 at the lumbar spine or total hip or femoral neck
- Women 65–69 years of age and men 70–79 years of age with T score values ≤ − 1.5 at the lumbar spine or total hip or femoral neck
- Postmenopausal women, men over 50 years of age with clinical risk factors
- Premenopausal women and younger men with a Z score < − 2 and having at least one specific risk factor:
  - History of fragility fracture of any site
  - History of height loss (shortening > 4 cm or prospective height shortening ≥ 2 cm)
  - Clinical findings of metabolic bone disease
  - Sustained back pain
  - Recent or ongoing usage of glucocorticoids and drugs related bone loss

#### Evaluation of the osteoporotic patient

An appropriate medical, clinical and laboratory evaluation is indicated in all adults who were diagnosed with OP, have an osteoporotic fracture history or have been identified as high risk given to coexisting medical conditions that contribute to bone loss. In addition, laboratory tests should be completed to exclude secondary causes of bone loss, which are often treatable. Table 3 summarizes secondary causes of osteoporosis in adults [21].

**Table 3:** Etiology of secondary osteoporosis in adults

Endocrine or metabolic causes	Nutritional/GI^a^ conditions	Drugs	Disorders of collagen metabolism	Other
Acromegaly Diabetes mellitus (types 1 and 2) Growth hormone deficiency Hypercortisolism Hyperparathyroidism Hyperthyroidism Porphyria Pregnancy Hypogonadism in men	Alcoholism Anorexia nervosa Calcium deficiency Chronic liver disease Malabsorption syndromes/malnutrition (celiac disease, cystic fibrosis, Chron’s disease, gastric resection) Total parenteral nutrition Vitamin D deficiency	Antiepileptics Aromatase inhibitors Chemotherapy/immunosuppressants Depo-Provera Glucocorticoids Gonadotropin-releasing hormone agents Heparin Lithium Proton pump inhibitors Selective serotonin reuptake inhibitors Thiazolidinedione Thyroid hormone (supraphysiologic doses)	Ehler-Danlos syndrome Homosisteinuria due to cystathionine deficiency Marfan syndrome Osteogenesis imperfecta	AIDS/HIV Ankylosing spondylitis Chronic obstructive pulmonary disease Gaucher disease Hemophilia Hypercalciuria Immobilization Major depression Myeloma and some cancers Organ transplantation Renal insufficiency/failure Rheumatoid arthritis Systemic mastocytosis Thalassemia

Adapted from American College of Endocrinology Clinical Practice Guidelines for the Diagnosis and Treatment of Postmenopausal Osteoporosis [21]

*AIDS*, acquired immune deficiency syndrome; *GI*, gastrointestinal; *HIV*, human immunodeficiency virus

The prevalence of secondary causes of OP is high in adults. Up to 30% of postmenopausal women and 50% of men with OP may have an underlying cause [28]. In men, secondary causes of OP are higher than in women [29].

Secondary causes for OP should be suspected in patients who present with a fragility fracture despite having no risk factors for OP and if the bone density Z score is < − 2. Baseline tests should be performed in every osteoporotic patient. Baseline and additional laboratory tests recommended by this panel are listed in Table 4 [21, 30]. If there is clinical suspicion of secondary OP, bone turnover markers (BTM) can be used for support diagnosis.

**Table 4:** Laboratory tests for OP

Baseline laboratory evaluation	
• Complete blood count (CBC) • Serum chemistry: calcium, phosphate, total protein, albumin, alkaline phosphatase, creatinine and electrolytes, liver enzymes (alanine aminotransferase (ALT), aspartate aminotransferase (AST), gamma-glutamyl transferase (GGT), bilirubin) • Serum 25-hydroxyvitamin D • 24-h urine collection for calcium^a^, sodium, and creatinine excretion • Total testosterone (in men)^b^ • Serum intact parathyroid hormone concentration	
Additional tests if clinically indicated might include (but not limited to):	
• Serum thyroid-stimulating hormone^c^ • Tissue transglutaminase antibodies for suspected celiac disease • Serum protein electrophoresis and free kappa and lambda light chains for suspected myeloma • Urinary free cortisol or other tests for suspected hypercortisolemia • Serum prolactin concentration • Bone turnover markers • Free testosterone • Serum tryptase, urine *N*-methylhistidine, or other tests for mastocytosis • Bone marrow aspiration and biopsy to look for marrow-based diseases • Undecalcified iliac crest bone biopsy with double tetracycline labeling • Genetic testing for rare metabolic bone diseases	

Adapted from American College of Endocrinology Clinical Practice Guidelines for the Diagnosis and Treatment of Postmenopausal Osteoporosis [21]

^a^The 24-h urine calcium collection must be done after vitamin D repletion if there is deficiency and under reasonable calcium intake (1000–1200 mg day^−1^) for at least 2 weeks

^b^As hypogonadism is a frequent cause of osteoporosis in men, serum total testosterone levels should be measured as baseline evaluation. If there is high suspicion of hypogonadism, further evaluation is required

^c^If the patient is receiving thyroid hormone replacement or suppression therapy or there is a clinical suspicion for hyperthyroidism, thyroid stimulating hormone should be measured

## Treatment of osteoporosis

### Calcium and vitamin D

Normal calcium and vitamin D (Vit D) status are crucial for maintaining bone metabolism and in the prevention and management of OP. The recommended dietary intake of calcium is between 800 and 1200 mg day^−1^, and there is no suggested dietary intake of Vit D [21]. Both supplements combined are recommended for patients at high risk of calcium and Vit D insufficiency, and those receiving anti-OP treatment [31, 32]. Additionally, adequate protein intake helps minimize bone loss and leads to better functional recovery after hip fracture [33].

### Dietary sources and supplements

Dietary sources of calcium are generally preferred over supplements. Calcium-rich foods are dairy products, beans, dark leafy vegetables, nuts, tofu, soy products, and fruit juices [34]. In Turkey, the most widely available calcium supplements are calcium carbonate and calcium citrate with differing amounts of elemental calcium content. Although calcium carbonate is the most affordable, its absorption is poor in the fasting state and is affected if the patient is taking proton pump inhibitors or H2 blockers. In general, supplements are not proposed in doses greater than 500 mg at a time because higher doses cause a plateau in calcium absorption [35]. Additionally, when calculating calcium intake, it should be advised that some foods, such as caffeine, soda, and high protein intake (> 2.0 g kg^−1^ day^−1^ [36]) may increase urinary calcium excretion or decrease its intestinal absorption [34]. This panel recommends a daily dietary calcium intake between 800 and 1200 mg for postmenopausal women and men above 50 years of age. Calcium supplementation should be given if the dietary intake is below 800 mg day^−1^. Furthermore, protein intake should be between 1.0 and 1.2 g kg^−1^ day^−1^, especially for elderly patients [37].

Vit D deficiency is a worldwide epidemic and is defined as serum 25(OH)D levels < 20 ng ml^−1^. Vit D insufficiency is defined as a serum 25(OH)D level of between 21 and 29 ng ml^−1^ [38]. Sources of Vit D in foods are extremely limited [39]. Although the main source of Vit D is sunlight exposure (UVB), it is not possible to achieve adequate Vit D levels exclusively from sunlight. Moreover, in subjects, who use sunscreen products, the synthesis of Vit D is limited. Additionally, Vit D production may not occur efficiently in the elderly [40]. In some countries, fortified dairy products are the major source of Vit D. However, in Turkey, this is not the case.

There are two forms of Vit D supplements: ergocalciferol (vitamin D_2_) and cholecalciferol (vitamin D_3_) [40]. Cholecalciferol should be the treatment of choice for Vit D deficiency or insufficiency since it increases serum 25(OH)D more efficiently than does ergocalciferol [41]. In the case of Vit D deficiency, 50,000 IU should be given orally weekly for 8 weeks as a loading dose and followed by a maintenance dose of 800 to 1500 IU day^−1^ orally [38]. A protective effect of Vit D on fractures and fall reduction was only seen at oral doses ≥ 800 IU day^−1^ [32]. Therefore, this panel recommends a daily oral dose of 800–1500 IU of cholecalciferol for postmenopausal women and men older than 50 years and at risk of fracture. However, it should be considered that no fracture benefit of Vit D alone is shown in recent meta-analyses [42]. For this reason, it is best to use calcium and Vit D together unless needing to treat Vit D deficiency.

### Supplement safety

Considering the widespread use of calcium and Vit D, the small risks of their side-effects (SEs) can translate into a large number of adverse events that can cause the individual to discontinue supplement usage [43–45]. A 17% relative risk increase for kidney stones with calcium and Vit D supplements has been reported in postmenopausal women [43]. Concerns have also been raised that calcium supplements may increase the risk of cardiovascular disease, but there is no sufficient evidence to demonstrate a significant association [46]. On the other hand, there are a few safety studies for Vit D. The safe upper level for Vit D is 4000 IU day^−1^ [47]. High doses of Vit D, particularly with calcium supplementation, can cause hypercalcemia, hypercalciuria, and kidney stones [48]. It is not advised to take large doses of Vit D (> 100,000 IU at a time), as it is associated with an increased risk of fracture and falls [48, 49].

### Pharmacological treatment options

Calcium and Vit D in the diet and supportive treatment alone are not sufficient for OP treatment; they should be used together with other agents to ensure healthy bone physiology. Antiosteoporotic drugs are recommended in patients with fragility fractures and in patients with fracture risk. Even though some of these drugs are approved, there are some reimbursement requirements in Turkey. Pharmacologic agents approved for the treatment of OP can be classified as either antiresorptive or anabolic. Each type of drug has shown to improve BMD and consequently reduce fractures (Table 5) [50].

**Table 5:** Pharmacological agents used for the treatment of OP

Drug	Treatment	Approval (by FDA)
Antiresorptive drugs		
Alendronate	10 mg PO daily^b^	PMO prevention and treatment
	70 mg PO weekly	GC OP
		Male OP
Risedronate	5 mg PO daily^b^	PMO prevention and treatment
	35 mg PO weekly	GC OP Treatment and prevention
	150 mg PO monthly	Male OP
Ibandronate	2.5 mg PO daily^b^	PMO prevention and treatment
	150 mg PO monthly	
	3 mg IV every 3 months	
Zoledronic acid	5 mg IV once yearly	PMO prevention and treatment
		GC OP prevention and treatment
		Male OP
		Prevention of new fracture after hip fracture
Denosumab	60 mg SC every 6 months	PMO treatment
		GC OP treatment^a^
		Male OP^a^
		Men with nonmetastatic prostate cancer receiving androgen deprivation therapy
		Women with breast cancer receiving aromatase inhibitor therapy
Raloxifene	60 mg PO daily	PMO treatment and prevention
		Prevention of breast cancer
Anabolic drugs		
Teriparatide	20 μg SQ daily	PMO treatment
		GC OP treatment
		Male OP
Abaloparatide^b^	Recently FDA Approved	
Romosozumab^b^	Recently FDA Approved	

*PO*, peroral; *SC*, subcutaneously; *IV*, intravenously

^a^Not reimbursed in Turkey

^b^Not available in Turkey

### Antiresorptive drugs

#### Bisphosphonates

Biphoponates (BPs) are the most commonly used antiresorptive agents and are usually the first line of choice when initiating treatment [51]. These drugs act by binding to bone-hydroxyapatite, accumulate in the bone [21, 52], differ significantly in terms of anti-remodeling potency, and the degree of persistence in the skeletal matrix [53]. Oral BPs are administered in weekly doses (alendronate and risedronate), monthly doses (ibandronate and risedronate), and intravenously (ibandronate and zoledronic acid).

Clinical experience indicates that BPs are generally safe. Oral and IV BPs are contraindicated in patients with hypocalcaemia, drug hypersensitivity to BPs, and severe renal impairment (GFR ≤ 35 ml min^−1^ for alendronate and zoledronate and ≤ 30 ml min^−1^ for other bisphosphonates) [21]. Rapid IV BPs may compromise kidney function, especially in older patients [54]. Atrial fibrillation due to zoledronate use has been reported as a serious SE but was later accepted as a coincidence [21]. SEs with the oral preparation include upper gastrointestinal (GI) issues, bowel disturbance, and muscle pain. IV administration may be associated with an acute phase reaction characterized by influenza-like symptoms, which typically occurs after the first injection [54].

Atypical femoral fractures and osteonecrosis of the jaw are rare but serious SEs. Risk for atypical femoral fractures is increased when BPs are used longer than 5 years [55]. In patients with dental disease or other related risk factors, dental examination is recommended prior to treatment. While undergoing treatment with BPs, patients should avoid invasive dental procedures, if possible. For patients requiring dental procedures, there is no data available to indicate whether discontinuation of treatment reduces the risk of osteonecrosis of the jaw [56].

#### Denosumab

Denosumab is a fully human monoclonal antibody and the first biologic agent approved for the treatment of OP. Denosumab blocks RANKL-RANK interaction reducing osteoclast formation, function, and survival and slows down bone reabsorption [57]. Denosumab is not accumulated in the bone tissue and is thought to be cleared from the bloodstream through the reticuloendothelial system [58]. Discontinuation of denosumab has been associated with an increase of bone turnover markers (BTMs) to above-baseline levels (rebound); these return to baseline within 2 years of treatment cessation. In parallel, BMD decreases to baseline levels within 1 to 2 years, regardless of the duration of previous therapy Therapy should be continued with another antiresorbtive to maintain the treatment efficacy.

Calcium deficiency, Vit D deficiency, and secondary hyperparathyroidism should be corrected prior to initiating denosumab treatment to avoid precipitating hypocalcemia. Rare cases of atypical femur fractures and osteonecrosis of the jaw have been observed with denosumab treatment. Denosumab can be used for patients with renal insufficiency but calcium must be monitored [21].

#### Raloxifene

Raloxifene is a selective estrogen-receptor modulator (SERM). It is a nonhormonal agent that has estrogen agonist effects in some tissues but could also have antagonist effects in others. Raloxifene is generally a safe and well tolerated agent. This drug is contraindicated in patients with venous thromboembolic disease, as venous thromboembolism is a rare but serious SE. Raloxifene should be discontinued 1 week before prolonged immobilization. Other SEs include mild to moderate hot flashes and leg cramps, peripheral edema, sweating, and endometrial fluid accumulation without endometrial disease [21].

### Anabolic agents

#### Teriparatide

Teriparatide (recombinant human parathyroid hormone 1–34) is a bone anabolic agent targeting cancellous bone. This drug induces a substantial increase in bone formation markers and, to a lesser degree, an increase in bone resorption markers [59]. It is contraindicated in hypermetabolic bone disease such as hyperparathyroidism and Paget’s disease of the bone, unexplained elevation of alkaline phosphatase, prior radiation therapy to the skeleton, severe renal impairment and patients with bone metastasis. Mild and transient SEs include nausea, orthostatic hypotension, and leg cramps. Hypercalcemia, while uncommon, is usually mild, asymptomatic, and transient [21]. Studies in rats have indicated an increased incidence of osteosarcoma [60], but these findings are not considered relevant for humans. The duration of treatment should not exceed 24 months.

#### Abaloparatide and romosozumab

Abaloparatide is a novel synthetic parathyroid hormone-related protein analogue [51]. Romosozumab is another novel treatment agent as a specific inhibitor of sclerostin [61]. These agents are not approved or available in the market in Turkey. However, these agents are available in other various countries.

### Combination therapy

Combination therapy is not recommended for the prevention or treatment of postmenopausal OP until its effect on fracture risk is demonstrated. Although no fracture data are available to support the combined use of anabolic/antiresorptive therapy, the combination of denosumab and teriparatide is promising in patients with the highest risk of fragility fractures [62]. A patient administered estrogen to treat menopausal symptoms or raloxifene to reduce the risk of breast cancer may also receive BP, denosumab, or teriparatide as an additional agent if they are at high risk for fractures [54].

### Sequential therapy

Sequential therapy, initiation of treatment with a bone-forming agent (teriparatide) and follow-up with antiresorptives may provide better long-term fracture prevention and should be the gold standard in patients with high risk of fractures [51]. In people with an increased risk of fracture, it is most reasonable to start treatment with an anabolic agent. Given that the effect of teriparatide treatment will disappear over time, the duration of the treatment should be limited to 24 months and continued with an antiresorptive drug. After denosumab treatment, therapy should be continued with BPs or raloxifene for at least 1 year to prevent further fractures [63].

## Treatment approach

Treatment of OP is dependent upon the risk level of the patient. This panel suggests Table 6 to define the risk levels and associated treatment options for OP.

**Table 6:** Risk levels of OP and associated treatments

High risk	
• Hip or recent spine fracture regardless of BMD • Multiple fragility fractures regardless of BMD • BMD T score of < − 2.5 and one vertebral fracture • The spine or hip BMD T score ≤ − 3.0 • Continuing hormone ablation therapy (e.g., aromatase inhibition, androgen deprivation therapy) and the spine or hip BMD T score ≤ −2.5 • Continuing glucocorticoid therapy and the spine or hip BMD T score ≤ − 2.5 • Age > 75 years and a T score < −2.5	
Treatment	
• First-line treatment is injectable pharmacological therapy (listed in alphabetical order): denosumab, teriparatide, zoledronic acid • Teriparatide may be the treatment of choice in patients with multiple vertebral fractures • Sequential therapy with anabolic followed by antiresorptive agents provides the greatest gain in BMD and will likely produce the greatest protection from long-term fracture risk	
Moderate risk	
• BMD T score in the range of − 2.5 to − 2.9, without fracture and any clinical risk factor • BMD T score ≤ − 1.0 and > − 2.5 with at least one clinical risk factor or any other osteoporotic fracture other than vertebra or hip	
Treatment	
• At any age, the treatment may be started with oral bisphosphonates (listed in alphabetical order): alendronate, risedronate • If the patient is < 65 years old, raloxifene or ibandronate may be recommended • For patients with contraindications to oral bisphosphonates or for those patients who do not prefer to have oral medications or if the clinician decides the patient cannot maintain oral therapy, injectable agents (denosumab, bisphosphonates) should be chosen	
Low risk	
• Middle aged women up to 65 years, BMD > − 2.5 at the hip and the lumbar spine with no other major risk factors • People who are not considered to be treated according to the Turkish FRAX model	
Treatment	
• Low-risk patients should be advised on taking calcium and vitamin D and to be physically active. Re-assessment is necessary at every 2–3 years.	

Although Turkish FRAX model was proposed theoretically, it has not been used in routine clinical practice throughout the country since the current reimbursement criteria allow prescription of anti-osteoporotic medication without using FRAX. Therefore, Turkey-specific FRAX model was not taken into account for the pharmacological treatment of osteoporosis in this consensus.

### Treatment duration

In the high-risk population with irreversible risk factors, life-long management of OP is usually necessary [64]. All non-BP medications produce temporary effects that wane following discontinuation [65] and maintenance of treatment benefits with other drugs may therefore often be required for certain agents [64]. BPs may have residual effects after treatment discontinuation which may be present for at least the following several years [65]. Below are the recommended treatment durations for osteoporotic agents:

- Treatment with teriparatide should be continued up to 2 years and should be followed by antiresorptive agents [21].
- Denosumab can be used continuously for up to 10 years. For patient which denosumab discontinuation is planned, sequential treatment with BP or raloxifene, in the case of BP, intolerance is recommended. Treatment with BP may be needed for up to 12–24 months [1].
- BP treatment is recommended for 3–5 years (3 years for zolendronic acid and 5 years for alendronate, ibandronate, and risendronate) followed by patient reassessment for fracture risk.
- Drug-holiday is recommended after 3 years of zolendronic acid and 5 years of oral BPs in patients with low fracture risk.
- Six years of zolendronic acid treatment and ten years of oral alendronate can be recommended in the following circumstances [1, 54, 64]:
  - Age 75 years or more
  - Previous history of hip or vertebral fracture
  - Occurrence of one or more low-trauma fractures during treatment (poor adherence and secondary OP should be excluded)
  - Current treatment with oral glucocorticoids ≥ 7.5 mg prednisolone day^−1^
  - Patients with high fracture risk based on clinical judgment or comorbidities
  - Persistently low hip BMD

There is no evidence that treatment beyond 10 years and management of such patients should be considered on an individual basis [54]. The duration of the treatment depends on the patient profile and the target to reach.

After treatment completion or discontinuation, or a new fracture, the fracture risk should be reevaluated regardless of the timing of new events. If no fracture occurs, measurement of BMD should be done within the next 18 months to 3 years [54]. Monitoring BTMs after 6 months and at regular intervals is recommended [66]. If available, C-telopeptide (CTX), aminoterminal propeptide of type 1 procollagen (PINP), and osteocalcin measurements are recommended as BTMs. Restarting treatment may be considered in patients who have a new fracture or significant BMD loss or increase of BTMs.

Given the length of most treatments, adherence to medication is a challenge when treating OP patients [67]. Interventions that are crucial for treatment adherence include education, improved communication, cultural respect, improvement in dosing programs, SE consideration, and appointment reminders [68].

## Challenges and recommendations for the management of OP in Turkey

### Challenges for OP Management in Turkey

Turkey is performing well in certain areas such as DXA access and uptake of FRAX. There is an estimated 13 DXA machines per million people available, indicating moderate availability [68] and these scans are reimbursed. The annual uptake of FRAX is high (1846 calculations per million of the population aged 50 years and over). Despite these areas of strong performance, a large treatment gap exists estimating that 75–90% of patients do not receive pharmacological intervention for secondary prevention. It has been reported that only one in five patients with hip fracture was previously diagnosed with OP and was on antiresorptive therapy [69]. Patients with OP are mainly managed by specialists in rehabilitation medicine, endocrinology, rheumatology, internal medicine, orthopedics, and gynecology.

OP is not officially accepted as a chronic disease. Therefore, it does not represent a public health priority and the cost of the pharmacological treatment is not completely reimbursed. This limitation interferes with the choice of medications physicians recommend to patients, possibly hindering beneficial outcomes. Furthermore, the restrictive reimbursement environment in Turkey is a significant barrier to appropriate OP management in the country.

The special authorization criteria required for reimbursement are extensive. Treatment of patients younger than 65 years of age is reimbursed if the T score is ≤ − 3.0. However, patients should be treated if the T score is ≤ − 2.5. FRAX is not taken into consideration for reimbursement even though there is a Turkey-specific FRAX model. Furthermore, denosumab is reimbursed only in female patients. For the reimbursement of teriparatide, the approval by an endocrinologist or a geriatrician is required even though OP patients are mostly managed by specialists in rehabilitation medicine.

There are a number of unmet needs in Turkey when assessing OP and a number of strategies to prevent the continual increase of the disease. Efforts should be made in various areas, including optimization of peak bone mass in young adults; implementation of four-step structural diagnostic procedures in patients with clinical risk factors for osteoporotic fractures (DXA, VFA, fall risk, and secondary OP); reduction in the treatment gap; management of OP through a multidimensional approach; and new strategies such as treat to target and definition of high-risk patients [70, 71]. Major low-cost targeted prevention strategies should be developed and implemented in Turkey to decrease the incidence of fractures, such as the following:

- Fall prevention program: a national program needs to be structured and implemented in order to prevent institutional and falls at home [72].
- Exercise program: a structured exercise program that includes walking, weight training, balance exercises, posture, and flexibility should be incorporated into the routines of the elderly [73, 74].
- Fracture liaison services (FLS): a coordinated care system that ensures individuals with fractures receive appropriate and multidisciplinary care should be implemented nation-wide [65].

### Recommendations to improve OP management in Turkey

Current demographics and prevalence of OP and related fractures in Turkey are silent but put constant pressure on the healthcare system. With this document, the panel expects to increase the awareness of this disease, decrease its consequences and the burden of OP in Turkey and make it a public health priority. To continue highlighting the importance of preventing, diagnosing and treating OP, this panel recommends:

- To the government and regulatory bodies:
  - Health authorities and other stakeholders should recognize OP as a major chronic disease ensuring the implementation of national awareness and prevention strategies.
  - Governmental bodies should reassess the restrictive reimbursement environment in Turkey.
  - The government should financially enable the creation of a national OP database to facilitate population-based research.
  - Health authorities and the government should financially support the establishment of OP prevention programs.
- To the scientific community:
  - Scientific societies and academic groups, with the support of the government and facilitated through the media, should strive to educate the at-risk population on OP through streamlined efforts.
  - Scientific and academic groups should develop online continuous medical education programs to encourage family practitioners to be a part of the continuum of care by identifying the at-risk patients and applying the already-existing standardized laboratory panels.
  - Scientific societies should encourage health care professionals to utilize the country-adjusted risk calculation algorithms.
  - Academic institutions should encourage researchers to pursue further investigations on country-based clinical and epidemiological data to facilitate the prevention and treatment of osteoporosis.

## Endorsements

The panel believes that as a result of implementing the above recommendations all adults will be better informed about the severity of osteoporosis, factors that may lead to osteoporosis, current recommendations and the fact that osteoporosis is, in fact, a major chronic disease affecting many adults in Turkey. As a result, we are confident that prevention of osteoporosis will improve greatly throughout Turkey.

These recommendations have been endorsed by:

- Turkish Osteoporosis Society
- The Society of Endocrinology and Metabolism of Turkey
- Turkish Society of Physical Medicine and Rehabilitation
- Turkish Menopause and Osteoporosis Society
- Turkish League Against Rheumatism
- Turkish Society for Rheumatology
- Turkish Geriatrics Society
